# Candida albicans Impacts Staphylococcus aureus Alpha-Toxin Production via Extracellular Alkalinization

**DOI:** 10.1128/mSphere.00780-19

**Published:** 2019-11-13

**Authors:** Olivia A. Todd, Mairi C. Noverr, Brian M. Peters

**Affiliations:** a Integrated Program in Biomedical Sciences, Microbiology, Immunology, and Biochemistry track, University of Tennessee Health Science Center, Memphis, Tennessee, USA; b Department of Microbiology and Immunology, Tulane University, New Orleans, Louisiana, USA; c Department of Clinical Pharmacy and Translational Science, University of Tennessee Health Science Center, Memphis, Tennessee, USA; d Department of Microbiology, Immunology, & Biochemistry, University of Tennessee Health Science Center, Memphis, Tennessee, USA; Carnegie Mellon University

**Keywords:** *Candida*, *Staphylococcus*, toxin, pH, polymicrobial, coculture

## Abstract

Candida albicans and Staphylococcus aureus are common causes of nosocomial infections with severe morbidity and mortality. Murine polymicrobial intra-abdominal infection (IAI) with C. albicans and S. aureus results in acute mortality dependent on the secreted cytolytic effector alpha-toxin. Here, we confirmed that alpha-toxin is elevated during polymicrobial growth compared to monomicrobial growth *in vitro*. Therefore, this study sought to unravel the mechanism by which C. albicans drives enhanced staphylococcal alpha-toxin production. Using a combination of functional and genetic approaches, we determined that an intact *agr* quorum sensing regulon is necessary for enhanced alpha-toxin production during coculture and that a secreted candidal factor likely is not implicated in elevating *agr* activation. As the *agr* system is pH sensitive, we observed that C. albicans raises the pH during polymicrobial growth and that this correlates with increased *agr* activity and alpha-toxin production. Modulation of the pH could predictably attenuate or activate *agr* activity during coculture. By using a C. albicans mutant deficient in alkalinization (*stp2*Δ/Δ), we confirmed that modulation of the extracellular pH by C. albicans can drive *agr* expression and toxin production. Additionally, the use of various *Candida* species (C. glabrata, C. dubliniensis, C. tropicalis, C. parapsilosis, and C. krusei) demonstrated that those capable of raising the extracellular pH correlated with elevated *agr* activity and alpha-toxin production during coculture. Overall, we demonstrate that alkalinization of the extracellular pH by the *Candida* species leads to sustained activation of the staphylococcal *agr* system.

**IMPORTANCE** Candida albicans and Staphylococcus aureus are commonly coisolated from central venous catheters and deep-seated infections, including intra-abdominal sepsis. Thus, they represent a significant cause of nosocomial morbidity and mortality. Yet how these organisms behave in the context of polymicrobial growth remains poorly understood. In this work, we set out to determine the mechanism by which activation of the staphylococcal *agr* quorum sensing system and production of its major virulence effector alpha-toxin is enhanced during coculture with C. albicans. Surprisingly, we likely ruled out that a secreted candidal factor drives this process. Instead, we demonstrated that alkalinization of the extracellular milieu by C. albicans and other *Candida* species correlated with elevated *agr* activity. Thus, we propose a mechanism where modulation of the extracellular pH by fungal opportunists can indirectly alter virulence of a bacterial pathogen. Uncovering molecular events that drive interkingdom pathogenicity mechanisms may enhance surveillance and treatment for devastating polymicrobial infections.

## Abstract (precis)

Candida albicans and Staphylococcus aureus are commonly coisolated from central venous catheters and deep-seated infections, including intra-abdominal sepsis. Thus, they represent a significant cause of nosocomial morbidity and mortality. Yet how these organisms behave in the context of polymicrobial growth remains poorly understood. In this work, we set out to determine the mechanism by which activation of the staphylococcal *agr* quorum sensing system and production of its major virulence effector alpha-toxin is enhanced during coculture with C. albicans. Surprisingly, we likely ruled out that a secreted candidal factor drives this process. Instead, we demonstrated that alkalinization of the extracellular milieu by C. albicans and other *Candida* species correlated with elevated *agr* activity. Thus, we propose a mechanism where modulation of the extracellular pH by fungal opportunists can indirectly alter virulence of a bacterial pathogen. Uncovering molecular events that drive interkingdom pathogenicity mechanisms may enhance surveillance and treatment for devastating polymicrobial infections.

## INTRODUCTION

Candida albicans, an opportunistic polymorphic fungus, and Staphylococcus aureus, a ubiquitous bacterial pathogen, rank among the top organisms responsible for life-threatening invasive disease. Not only do these pathogens cause significant morbidity and mortality on their own, evidence for their existence as part of polymicrobial consortia has surfaced. For instance, C. albicans and S. aureus have been coisolated from a variety of biotic and abiotic surfaces, including central venous catheters, prosthetic implants, skin, and mucosal layers (1, 2). Moreover, they have been coassociated with several polymicrobial diseases, including burn wound superinfection, ventilator-associated pneumonia, urinary tract infection, cystic fibrosis, and bloodstream infection (3–6). However, perhaps the most comprehensive line of investigation of this particular coinfection has focused on their role during intra-abdominal infection (IAI).

IAI is a spectrum of diseases characterized by microbial invasion and subsequent inflammation of the abdominal cavity (7). Mortality rates for such infections typically range between 10 and 30%; however, mortality rates involving a fungal pathogen (e.g., C. albicans) can approach 80%, even with appropriate treatment (8, 9). Using a murine model of IAI, a series of studies by Carlson demonstrated that C. albicans enhanced the virulence of S. aureus, as coinfection reached 100% mortality within days postinfection (p.i.) while monomicrobial infection with either pathogen was nonlethal (10). Studies designed to titrate various inoculating doses of C. albicans and S. aureus during coinfection revealed that this apparent synergism was not mutual, as C. albicans virulence was not augmented by low doses of S. aureus (11). Synergistic mortality rates were found to be dependent on various toxins produced by S. aureus (12). Although these studies failed to delineate which specific S. aureus toxin was responsible, they established their important role in driving pathogenicity in the context of polymicrobial IAI.

Toxin expression in S. aureus is governed by a complex set of transcriptional regulators that respond to endogenous and environmental stimuli. Perhaps the best well-characterized mechanism is driven by the accessory gene regulator (*agr*) quorum sensing system that is activated in a cell density-dependent manner. The *agr* operon is composed of four genes encoding AgrA, AgrB, AgrC, and AgrD proteins (13). AgrD serves as the immature signal peptide that is proteolytically processed and secreted by membrane-bound AgrB, releasing the mature signal molecule autoinducing peptide 2 (AIP2). AIP2 can be sensed by the surface-bound receptor and histidine kinase AgrC that phosphorylates and activates the AgrA transcription factor. Activation of AgrA simultaneously upregulates expression of the *agr* operon completing a positive-feedback loop, while also ultimately downregulating colonization factors (e.g., adhesins) and upregulating virulence factors, including toxins (13, 14). While *agr* is undoubtedly induced as a consequence of quorum development, it is also highly susceptible to environmental factors, including high salt, glucose, subinhibitory antibiotic concentrations, and pH (15, 16).

Our laboratory has recently demonstrated that during *in vitro* growth, C. albicans has the capacity to activate the *agr* regulon, leading to exacerbated production of alpha-toxin, a potent staphylococcal virulence determinant capable of lysing a variety of host cells, causing tight-junction loss, and activating numerous innate proinflammatory pathways (17). Moreover, this virulence factor was crucial for driving lethal synergism during polymicrobial IAI. By using a combination of genetic and functional assays, the objective of this study was to attempt to elucidate the mechanism by which C. albicans activates *agr* signaling and alpha-toxin production by S. aureus and to determine the extent that other non-*albicans Candida* (NAC) species can augment alpha-toxin release (17). In the course of conducting these studies, we discovered that modulation of the extracellular pH by C. albicans creates an optimal environment for robust induction of the staphylococcal *agr* system, further highlighting how complex ecological signals may intersect with virulence during this prevalent fungal-bacterial interaction.

## RESULTS

### C. albicans augments staphylococcal alpha-toxin production and does not complement *agr* signaling in *trans*.

**FIG 1:**
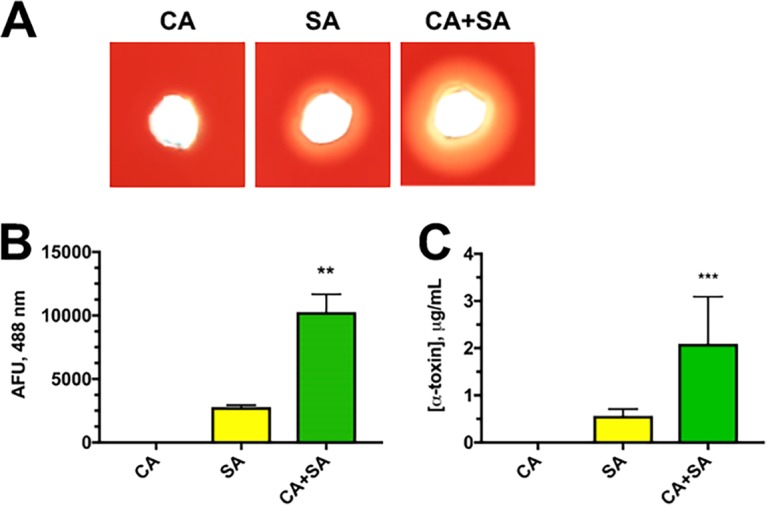
C. albicans enhances S. aureus alpha-toxin production. (A) Hemolytic activity of monomicrobial (C. albicans SC5314 [CA] or S. aureus JE2 [SA]) and polymicrobial cultures (CA plus SA [CA+SA]) was functionally assessed by depositing 20 μl of cell-free culture supernatants into wells on a sheep blood agar plate. Images were taken after incubation at 37°C for 24 h using a digital scanner. (B) A P3-GFP reporter strain of S. aureus was incubated alone or with C. albicans. After 16 h of growth, 100 μl culture was removed in triplicate and added to a 96-well plate, and fluorescence (in arbitrary fluorescence units [AFU]) was measured at 488/515 nm on a fluorimeter. (C) The concentration of alpha-toxin in supernatants from monomicrobial and polymicrobial cultures was measured by ELISA. Data are representative of three independent repeats and expressed as the means plus standard errors of the means (SEM) (error bars). Data were assessed for significance using one-way analysis of variance (ANOVA) and Dunnet’s posttest. Values that are significantly different are indicated by asterisks as follows: **, *P* < 0.01; ***, *P* < 0.001.

Similar to previously reported findings (17), coculture of C. albicans and S. aureus led to elevated hemolytic toxin production compared to monoculture (Fig. 1A), as assessed by a functional hemolytic assay on sheep blood agar. Unsurprisingly, monoculture of C. albicans did not demonstrate lysis (Fig. 1A), as this fungus is not commonly reported to lyse red blood cells on microbiological agar. While the regulation of virulence factor production in S. aureus is multifactorial, the *agr* quorum sensing system plays a major role in governing increased toxin expression. In order to confirm that the *agr* system was more robustly activated during coculture, a P3-GFP (green fluorescent protein) reporter system (the P3 promoter is a target of phosphorylated and activated AgrA and ultimate driver of toxin expression) was employed. Indeed, the results of reporter analysis indicated an approximately 2.5-fold induction of *agr* (Fig. 1B), which correlated with ∼4-fold production of alpha-toxin as measured by a specific enzyme-linked immunosorbent assay (ELISA) (Fig. 1C). These results were consistent with those in Fig. 1A, given that the hemolytic phenotype observed on sheep blood agar is dependent on alpha-toxin activity.

**FIG 2:**
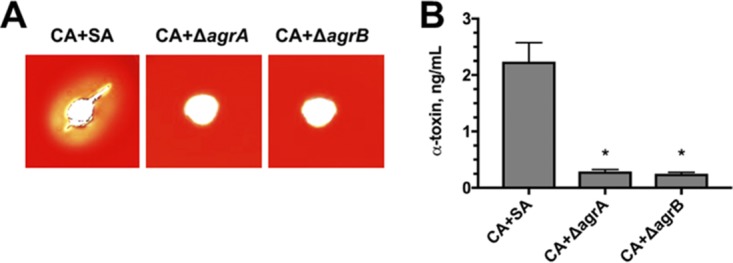
*Candida*-enhanced toxin production is dependent on intact staphylococcal *agrA* and *agrB* signaling. (A) Hemolytic activity of polymicrobial cultures of C. albicans (CA) with wild-type S. aureus (SA) or isogenic strains lacking *agr* genes (Δ*agrA* or Δ*agrB*) was functionally assessed by depositing 20 μl of cell-free culture supernatants into wells on a sheep blood agar plate. Images were taken after incubation at 37°C for 24 h using a digital scanner. (B) Levels of alpha-toxin were measured in polymicrobial culture supernatants by ELISA. Data are representative of three independent repeats and expressed as the means plus SEM. Data were assessed for significance using one-way ANOVA and Dunnet’s posttest. Statistical significance: *, *P* < 0.05.

The next logical line of investigation was to determine whether C. albicans may be producing a protein or other small molecule that could be activating the *agr* system in S. aureus, leading to upregulation of toxin. Loss of *agrA* is predicted to largely ablate quorum sensing and elevated toxin production, as its activated form binds to both P2 and P3 promoters to drive the *agr* regulon and decrease repressor of toxin (*rot*), respectively. However, deletion of *agrB* would theoretically only attenuate secretion of the quorum signal peptide autoinducing peptide 2 (AIP2) (18–20). During monoculture, this would disrupt sensing of the quorum signal via AgrC and negatively impact toxin production. However, if C. albicans produced a molecule(s) that could be sensed by AgrC, then the native regulatory circuit could be bypassed during coculture, resulting in elevated toxin expression. Therefore, similar coculture assays were conducted as in Fig. 1, this time including isogenic Δ*agrA* and Δ*agrB* mutants. As predicted, disruption of *agrA* led to a nearly complete loss of hemolysis on sheep blood (Fig. 2A) and significantly reduced levels of alpha-toxin (Fig. 2B). Moreover, deletion of *agrB* demonstrated similar toxin phenotypes compared to the Δ*agrA* mutant (Fig. 2A and B). These results suggested that an intact *agr* regulon is required for elevated toxin expression. However, the augmented toxin phenotype was unlikely mediated by a fungal ligand-bacterial receptor interaction governed through AgrC sensing.

### A role for extracellular pH in modulating *agr* signaling during coculture.

**FIG 3:**
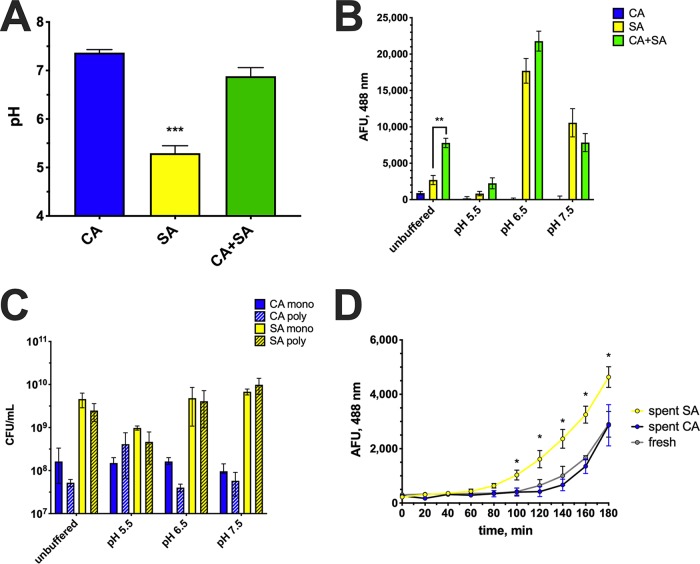
Synergistic hemolysis is partially dependent on extracellular pH and can be overridden by its modulation. (A) The pH of monomicrobial (SA or CA) and polymicrobial (CA+SA) cultures was measured after 16 h of incubation at 37°C. (B) Assays were conducted similarly as in panel A, except that the pH of TSB-g was buffered as indicated. Activation of staphylococcal *agr* was assessed using a GFP reporter assay. (C) Aliquots from each culture were taken at the endpoint to measure microbial counts by plating on selective media. Blue bars indicate C. albicans counts, and yellow bars indicate S. aureus counts. Solid color bars depict counts from monomicrobial (mono) cultures, while hatched bars depict counts from polymicrobial (poly) cultures. (D) Buffered (pH 7.0) spent CA and SA culture supernatants or fresh media were added to S. aureus(pDB22) initially grown at pH 5.5. Fluorescence (488 nm) was captured kinetically to assess *agr* activation. All data are representative of three independent repeats and expressed as the means ± SEM. Data were assessed for significance using one-way ANOVA and Dunnet’s posttest. *, *P* < 0.05; **, *P* < 0.01; ***, *P* < 0.001.

Previous reports have determined that regulation of *agr* is influenced by a number of physiologic factors, including low extracellular pH (15, 16). Therefore, we assessed the pH of monocultures and cocultures following standard growth conditions. The pH of C. albicans monoculture was estimated to be ∼7.5 ± 0.1, while that of S. aureus monoculture was ∼5.2 ± 0.2 (Fig. 3A). Interestingly, the pH of the coculture was ∼6.7 ± 0.2 (Fig. 3A). The pH of fresh Trypticase soy broth (TSB) plus 0.2% glucose (TSB-g) was determined to be 7.2 ± 0.2 (data not shown). Therefore, it appeared that C. albicans was elevating or maintaining the pH in a range which is optimal for *agr* activation. We next determined whether enhanced *agr* activity during coculture could be overridden by experimental modulation of the pH via buffering TSB-g with 100 mM morpholinepropanesulfonic acid (MOPS). We specifically chose to assess pH points that closely matched those representing C. albicans monoculture, S. aureus monoculture, and coculture values. Similar to previous results, use of the P3-GFP reporter system indicated induction of *agr* signaling in unbuffered medium (Fig. 3B). However, when the pH was buffered to acidic conditions (pH 5.5), *agr* signaling was significantly attenuated (Fig. 3B). When the pH was set to the *agr* optimum (pH 6.5), much higher levels of *agr* signaling were observed; however, differences between mono and coculture were no longer distinguishable (Fig. 3B). Similar results were found at a slightly more alkaline pH (pH 7.5), where *agr* signaling between monoculture and cocultures was indiscernible (Fig. 3B). These results suggest that extracellular pH strongly influences *agr* signaling *in vitro* and likely drives augmentation of toxin production during coculture.

In order to rule out the possibility that growth rates between different pH conditions were impacting *agr* reporter results, aliquots of each culture were taken at the experimental endpoint and plated onto selective microbiological media to enumerate both C. albicans and S. aureus. Although there was slight variation between each condition, there was no significant difference in the number of colonies of C. albicans (Fig. 3C) recovered during monoculture and coculture. Moreover, there was no significant difference between growth in buffered or unbuffered media. The same finding was true for S. aureus (Fig. 3C) during monoculture and coculture. Collectively, these results suggest that extracellular pH and not microbial growth accounts for disparate *agr* activity observed during mono- and coculture.

**FIG 4:**
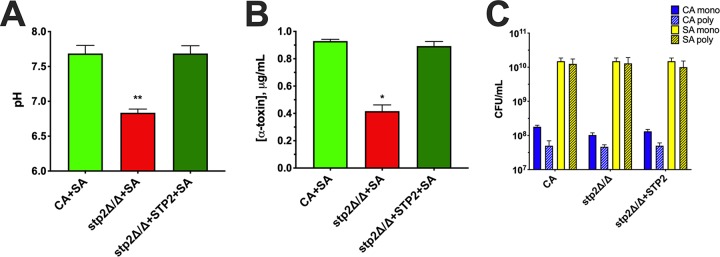
The alkalinization-deficient C. albicans *stp2*Δ/Δ mutant fails to enhance S. aureus toxin production. Polymicrobial cultures of S. aureus and wild-type C. albicans (CA+SA), SA and *stp2* mutant (*stp2*Δ/Δ+SA), or SA and *STP2* revertant (*stp2*Δ/Δ+*STP2*+SA) were incubated at 37°C in media with an initial pH of 6. At 24 h, the pH of the cultures were measured using a pH meter (A), and alpha-toxin levels in supernatants were determined by ELISA (B). (C) Aliquots from each culture were taken at the endpoint to measure microbial counts by plating on selective media. Blue bars indicate C. albicans counts, and yellow bars indicate S. aureus counts. Solid color bars depict counts from monomicrobial (mono) cultures, while hatched bars depict counts from polymicrobial (poly) cultures. Data are representative of three independent repeats and expressed as the means plus SEM. Data were assessed for significance using one-way ANOVA and Dunnet’s posttest. *, *P* < 0.05; **, *P* < 0.01.

In order to rule out the possibility that a factor secreted by C. albicans activates *agr* signaling, S. aureus(pDB22) was grown at pH 5.5 overnight to attenuate *agr* signaling. Spent culture supernatants from C. albicans or S. aureus JE2 grown at pH 7 were filter sterilized, reconstituted in fresh concentrated growth medium, and added to S. aureus(pDB22) to elicit *agr* activation. Fresh culture medium was also used as a control. Results demonstrated that while spent culture medium from strain JE2 more rapidly and robustly activated the *agr* system, the addition of spent C. albicans supernatant did not give results different from those after the addition of fresh culture medium, suggesting that culture pH and not a specific candidal factor drives quorum signaling (Fig. 4D).

Several recent reports have demonstrated that C. albicans possesses the incredible capacity to rapidly alkalinize its external environment via amino acid catabolism (21–23). This process is primarily driven during carbohydrate stress by sensing of amino acids in the milieu and regulating amino acid import, largely governed by transcription factor Stp2p. Ammonia is extruded from the cell as these peptide substrates are consumed by the fungus, ultimately raising the extracellular pH (24, 25). Therefore, we utilized an *stp2*Δ/Δ mutant and isogenic revertant strain (*stp2*Δ/Δ+*STP2*) to determine the impact of alkalinization during coculture with S. aureus. The culture pH of the *stp2*Δ/Δ mutant during coculture was significantly decreased compared to that of the wild-type or revertant strains (Fig. 4A). In a similar fashion, production of alpha-toxin was significantly attenuated during coculture with the *stp2*Δ/Δ strain (Fig. 4B). Thus, active alkalinization of the external environment by C. albicans partially modulates staphylococcal alpha-toxin production during coculture.

### *Candida* species differentially modulate alpha-toxin production during coculture.

**FIG 5:**
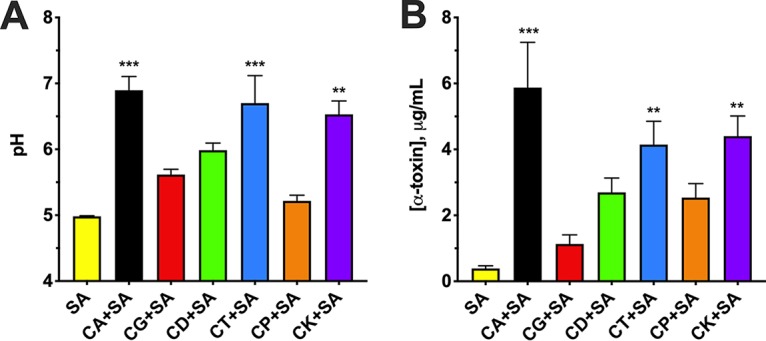
*Candida* spp. differ in their capacity to modulate extracellular pH and augment toxin production during polymicrobial growth with S. aureus. Monomicrobial or polymicrobial cultures of *Candida* species (C. albicans [CA], C. glabrata [CG], C. dubliniensis [CD], C. tropicalis [CT], C. parapsilosis [CP], and C. krusei [CK]) and S. aureus (SA) were incubated at 37°C. After 16 h of growth, the pH of the cultures was measured using a standard pH meter (A), and the level of alpha-toxin in the supernatant was assessed by ELISA (B). Data are representative of five independent repeats and expressed as the means plus SEM. Data were assessed for significance using one-way ANOVA and Dunnet’s posttest. **, *P* < 0.01; *** *P* < 0.001.

We next questioned whether augmented alpha-toxin production was specific to C. albicans or whether other non-*albicans Candida* (NAC) species could also potentiate this effect. Therefore, S. aureus was cultivated by itself or in the presence of various *Candida* species, including C. albicans, C. glabrata, C. dubliniensis, C. tropicalis, C. parapsilosis, and C. krusei. Measurement of the extracellular pH following coculture with C. albicans, C. tropicalis, and C. krusei revealed significantly increased neutralization compared to S. aureus monoculture (Fig. 5A). Coculture with C. glabrata, C. dubliniensis, and C. parapsilosis demonstrated only modest increases in pH which were generally below the threshold for robust *agr* activation. These results were recapitulated by examining alpha-toxin production during coculture. *Candida* species capable of significantly raising the extracellular pH in this assay (C. albicans, C. tropicalis, and C. krusei) also demonstrated increased capacity to augment alpha-toxin release, while those incapable of significantly modulating the extracellular pH demonstrated only modest elevation of this virulence determinant (Fig. 5B). These results demonstrate that exacerbation of alpha-toxin production is not limited to C. albicans, but this *in vitro* phenotype is largely driven by modulation of extracellular pH during coculture.

## DISCUSSION

Often, the pathogenic process is delineated as the interaction between host and microbe resulting in some level of host detriment. Unfortunately, the contribution of microenvironment is all too frequently disregarded in this description. However, changes in environmental factors can ultimately drive or control pathogenicity or host susceptibility. Through the prism of single microbe infections, this concept seems obvious. For example, gene expression exhibited by C. albicans is very different in the gut compared to in the oral cavity—largely attributable to variations in host cell type, oxygen tension, nutrient availability, and microbial competition (26–28). In the context of a polymicrobial model of disease, gene regulation in response to environment by one organism may elicit reactions by a second pathogen, opportunist, or colonizer. An excellent example of this is the capacity of the lactobacilli to ferment carbohydrates to lactic acid at the vaginal mucosa (29). Production of relatively high levels of lactic acid keeps the vaginal pH low and consequently impairs the overgrowth of C. albicans, limiting the development of vulvovaginal candidiasis. While there are several examples of “environmental cross talk” resulting in microbial antagonism, reports regarding exacerbated virulence are less common (30–33).

We, along with others, have previously shown that murine intra-abdominal coinfection with C. albicans and S. aureus results in a strikingly high mortality rate (∼90%) within ∼16 h postinfection (p.i.), while infection with either microbe alone is nonlethal (17). Moreover, this synergistic lethality is dependent on alpha-toxin, as an isogenic strain lacking the gene encoding this virulence factor (*hla*) or neutralization of this toxin using a high-affinity antibody resulted in significant protection. In support of this, lavage of the peritoneal cavity following infection revealed ∼4-fold-higher levels of alpha-toxin in coinfected mice compared to mice challenged with S. aureus alone (17). These *in vivo* results substantially mimic those observed in the *in vitro* system used in this study.

Using several functional assays, we confirmed that alpha-toxin levels are significantly elevated during polymicrobial growth compared to monomicrobial growth in an *agr*-dependent fashion. Interestingly, the mechanism of alpha-toxin enhancement was not likely due to direct fungal ligand binding or recognition by the *agr* quorum sensing system, as S. aureus mutants with disrupted *agr* genes (Δ*agrA* or Δ*agrB*) failed to demonstrate toxin production even during coculture with C. albicans, indicating the necessity of an intact *agr* regulon (Fig. 2). There are numerous examples where secreted microbial peptides or metabolites can exhibit a diverse array of effects on neighboring cells of the same or different species (30, 33, 34). Deletion of *agrB*, the autoinducing peptide permease, should still allow for functional *agr* signaling in the context of candidal factors capable of inducing the AgrC-AgrA two-component system. The observed failure to activate toxigenic and *agr* responses under such conditions or with spent C. albicans culture supernatant likely indicates that potential fungal ligands do not directly signal via the AgrC surface receptor.

The exclusion of a factor directly engaging *agr* led us to investigate potential indirect influences, such as modulation of environmental factors. In pioneering work characterizing the *agr* quorum sensing system in S. aureus, Regassa et al. demonstrated the pH responsiveness of the *agr* system. They reported increased *agr* activity during growth at pH 6.5 to 7 compared to growth in more alkaline or acidic media (15, 16). The pH of monomicrobial S. aureus cultures demonstrated significant acidification (pH 5.0 to 5.5), while cocultures exhibited a pH of 6.5 to 7, exactly in the range of maximum *agr* activity (Fig. 3A). Buffering of the media demonstrated that *agr* signaling could be manipulated positively or negatively during coculture depending on pH selection, further demonstrating the importance of environmental factors (e.g., pH) in driving pathogenicity mechanisms *in vitro*. Both C. albicans and S. aureus preferentially utilize glucose to undergo oxidative and fermentative metabolism, ultimately producing acidic end products that drive lower culture pH. So then why does the culture medium containing C. albicans demonstrate an elevated pH? The ability of C. albicans (and other fungal species) to alkalinize its environment has been well documented in the literature. Vylkova and Lorenz have demonstrated that C. albicans can raise the pH of macrophage phagosomes, allowing for hyphal growth that damages the phagosomal membrane, aiding in escape and continued proliferation (21, 22). Moreover, the acidic pH (≤5) of the phagosome is vital for the activity of degradative enzymes that act to kill and digest engulfed pathogens, including fungi. Additionally, low pH is known to repress the morphological transition of yeast to hyphae, the major virulence attribute of C. albicans (35). Therefore, C. albicans has likely evolved strategies to modulate the external pH to bypass host checkpoints and killing mechanisms.

We propose that during polymicrobial growth, the pH initially decreases as glucose is metabolized by both organisms, then when glucose is limiting, the culture medium is alkalinized by C. albicans, in turn activating the *agr* quorum sensing system and subsequent alpha-toxin production. The mechanism of alkalinization involves the breakdown of amino acids for a carbon source and the subsequent excretion of ammonia, which raises the pH. In glucose-limited environments, amino acids are sensed by the SPS sensor system, a complex of three proteins (Ssy1, Ptr3, and Ssy5). This sensor complex induces the proteolytic cleavage of a cytoplasmic retention signal of Stp2p, allowing it to translocate to the nucleus. Stp2p binds SPS sensor-regulated promoters of various amino acid permease genes, including *CAN1*, *GAP1*, and *GAP2*, which transport extracellular amino acids into the cell (36). Amino acid catabolism begins with the deamination of an amino acid, catalyzed by amino acid-specific deaminases. The carbon backbone is converted to tricarboxylic acid (TCA) cycle intermediates (pyruvate, α-ketoglutarate, and acetoacetyl coenzyme A [acetoacetyl-CoA]) via the production of acetyl-CoA. The nitrogen is converted to ammonia and CO_2_ by the urea amidolyase Dur1,2p, and subsequently excreted from the cell through various Ato (ammonia transport outward) family transmembrane proteins (23, 25, 36). C. albicans strains lacking *STP2* have an impaired capacity to alkalinize the environment compared to isogenic controls, corresponding with a decrease in ammonia produced during growth. Additionally, this mutant is unable to form hyphae after phagocytosis, preventing escape from the phagosome, which also leads to more effective killing by the macrophage. In a mouse model of disseminated candidiasis, *stp2*Δ/Δ mutants displayed attenuated virulence as mortality was significantly delayed compared to wild-type or complemented strains (22). Using a *stp2*Δ/Δ strain in our *in vitro* polymicrobial culture system, we found that the mutant cannot raise the pH to levels observed with the wild-type or revertant strains. This further confirms that the capacity of C. albicans to alkalinize the media contributes to enhanced alpha-toxin production during coculture (Fig. 4).

Although Stp2p is largely responsible for driving the alkalinization phenotype in C. albicans, it is not the sole mechanism for pH modulation. A number of C. albicans genes have been identified as having effects on external alkalinization, including *ALI1*, *SIN3*, *COX4*, *PEP8*, *KIS1*, and *CPH1*. Some of these genes (*COX4* and *KIS1*) are linked to carbon metabolism, while *CPH1* can regulate galactose utilization. C. albicans has also been shown to modulate pH without the production and excretion of ammonia, as seen during growth with nonfermentable carbon sources, like the carboxylic acids α-ketoglutarate, pyruvate, and lactate (24, 37). Interestingly, alkalinization occurs in low-glucose environments, regardless of the mechanism, indicating that this effect is glucose repressible. Vylkova and Lorenz hypothesize that alkalinization of the phagosome is due to limited glucose within the phagosomal compartment (22). Although glucose or other metabolic compounds were not a direct focus of this paper, their potential effect on pH and alpha-toxin production can be inferred. In addition to being responsive to pH, the *agr* quorum sensing system is known to be regulated by glucose levels, with growth under high glucose conditions correlated to low *agr* activity. Future studies focusing on the metabolic profiles of both C. albicans and S. aureus may shed more light on this potential effector mechanism.

Last, we investigated the ability of other NAC species to enhance alpha-toxin production during *in vitro* growth. We found that the species differ in their alkalinization potential, with only C. tropicalis and C. krusei able to raise the pH of the media to levels similar to that of C. albicans during polymicrobial growth with S. aureus. Additionally, these three species were the only ones that caused a significant increase in the amount of alpha-toxin produced compared to S. aureus monomicrobial culture (Fig. 5). These data correlate nicely with work performed by Nash et al. in which mice were coinfected with the exact same *Candida* strains. Mortality was observed in mice infected with S. aureus and C. albicans, C. tropicalis, and C. krusei. Strikingly, the species unable to modulate the pH and alpha-toxin production during *in vitro* growth in this study (C. glabrata, C. dubliniensis, and C. parapsilosis) were also nonlethal in the polymicrobial IAI model (38). A retrospective analysis of intra-abdominal infections, specifically looking at candidiasis, revealed that C. albicans is the most commonly isolated species (50 to 75%). The next most commonly isolated species is C. glabrata (12 to 25%), followed by C. parapsilosis (3 to 10%), C. tropicalis (3 to 5%), and C. krusei (3 to 6%) (39, 40). Interestingly, C. glabrata was implicated as the infectious organism in the majority (64%) of recurrent or persistent infections (39). Cheng et al. described persistent C. glabrata IAI in mice and an association with the formation of abscesses (41).

Various niches within the body are maintained at drastically different pH values. For example, the oral cavity is maintained at pH 6.2 to 7.6 by saliva (42). Blood is very tightly buffered to remain at pH 7.4, and a decrease in 0.05 units causes severe physiological problems, as seen in diabetic ketoacidosis (43). The vagina is more acidic, with a healthy pH ranging from 3.8 to 4.5 (44). The fluid within the peritoneal cavity of humans is reported to be at a pH of 7.5 to 8 (45). Although widely variable, bodily pH is tightly regulated to maintain homeostasis, and dysregulation is often indicative of poor health. A study investigating the transcriptome of C. albicans during murine IAI found that a number of genes involved in pH response were among the most highly upregulated. These genes include *RIM101*, the alkaline pH-regulated transcription factor that modulates morphology and gene expression (26). Indeed, a *rim101*Δ/Δ strain exhibited attenuated virulence during peritoneal infection, characterized by significantly lower fungal burdens (26). These findings indicate that the murine peritoneal cavity is alkaline and that C. albicans requires adaptation to pH to establish pathogenicity in this biological niche. However, because of the homeostatic nature of pH maintenance, it is unlikely that that C. albicans is able to drastically alter the global pH of the peritoneal cavity during IAI. Our attempts to experimentally monitor broad pH changes in the peritoneal lavage fluid during mono- or coinfection have demonstrated insignificant differences, partially due to sensitivity of the techniques utilized (e.g., phenol red lavage, micro pH electrode) or spatiotemporal kinetics (data not shown). Thus, it is more likely that microenvironmental pH regulation, in peritoneal abscesses or tissues, may play an important role in driving these phenotypes. As C. albicans and S. aureus are known to tightly associate via staphylococcal binding of fungal hyphae via the candidal adhesin Als3p (46), it is possible that elevated S. aureus alpha-toxin production *in vivo* is due to local pH changes surrounding fungal-bacterial aggregates. However, this hypothesis requires future investigation.

Collectively, results from this study highlight the dynamic and complex nature of this fascinating microbial pair and polymicrobial interactions in general. Furthermore, they underscore the importance of environmental adaptation and its intersection with virulence that must be considered in the context of coinfection.

## MATERIALS AND METHODS

### Strains and growth conditions.

Candida albicans SC5314 (referred to as CA) was used as the wild-type isolate throughout this work. The alkalinization-deficient mutant *stp2*Δ/Δ (SVC17) and its isogenic revertant strain *stp2*Δ/Δ+*STP2* (SVC19) were kind gifts from Michael Lorenz (University of Texas Health Science Center) (22). The non-*albicans Candida* (NAC) species C. glabrata CBS138 (CG), C. dubliniensis CD36 (CD), C. parapsilosis CDC317 (CP), C. tropicalis MYA3404 (CT), and C. krusei 81-B-5 (CK) were used as species-representative strains (38). JE2, a USA300 isolate, was obtained from the Biodefense and Emerging Infectious (BEI) Research Resources repository and used as the wild-type S. aureus strain in this work and is referred to as SA. A S. aureus reporter strain [S. aureus(pDB22)] containing plasmid pDB22 (containing the P3 promoter fused to GFP_mut2_ and an erythromycin resistance cassette) was also used in this work (47). All strains were maintained as 20% glycerol stocks at –80°C.

*Candida* strains were streaked onto yeast-peptone-dextrose (YPD) agar and grown at 30°C. A single colony was inoculated in YPD broth and incubated overnight at 30°C with shaking at 200 rpm. S. aureus strains were streaked on Trypticase soy agar (TSA) (with 10 μg/ml erythromycin added as needed). Single colonies were inoculated in Trypticase soy broth (TSB) and grown overnight at 37°C with shaking at 200 rpm. Aliquots (500 μl) were washed three times with phosphate-buffered saline (PBS), and cell concentrations were adjusted to 1 × 10^7^ CFU/ml. A 1:100 dilution was made into 5 ml of 0.6× TSB plus 0.2% glucose (TSB-g) with the following groups: CA or SA (monomicrobial) and CA plus SA (CA+SA) (polymicrobial). PBS (50 μl) was added to monomicrobial cultures. Cultures were incubated at 37°C with shaking at 200 rpm, and aliquots were removed at 16 h postinoculation (p.i.). Mono- and polymicrobial cultures using the alkalinization-deficient mutant and revertant strains were prepared as described above, except TSB-g was adjusted to pH 6 prior to inoculation and cultures were incubated for 24 h.

### Agr reporter assay.

Monomicrobial and polymicrobial cultures were prepared as described above using CA and/or S. aureus(pDB22) (10 μg/ml erythromycin added for plasmid maintenance). At 16 h p.i., 100-μl aliquots were removed in triplicate from cultures and added to wells of 96-well black microtiter plate. Fluorescence (488 nm excitation, 525 nm emission) was measured using a plate reader (Synergy, Biotek). Experiments were repeated in triplicate, and results are expressed as the mean arbitrary fluorescence units (AFU) ± standard errors of the means (SEM).

### Blood agar lysis assay.

Cultures were prepared as described above, and at 16 h p.i., 5-ml aliquots were centrifuged at 5,000 rpm to pellet cells, and supernatants were sterilized using 0.2-μm syringe filters. Sterile supernatants were concentrated 20× by ethanol precipitation. Holes were punched in blood agar plates (TSA with 5% sheep blood) using a sterile pipette tip. Concentrated supernatant (20 μl) was added to wells, and plates were incubated at 37°C for 24 h. Plates were photographed using a digital scanner (EPSON Perfection V700 Photo).

### pH buffering.

pH buffering experiments were done by the same culture set-up described above, with the following changes: 100 mM morpholinepropanesulfonic acid (MOPS) was added to 0.6× TSB plus 0.2% glucose, and the medium was adjusted to various pHs (5.5, 6, 6.5, 7,7.5, and 8) using 5 N HCl or NaOH as required. Aliquots (500 μl) of overnight cultures of CA and S. aureus(pDB22) were washed with PBS and adjusted to 1 × 10^7^ CFU/ml. A 1:100 dilution was made into 5 ml MOPS-buffered TSBg at each pH tested. Monomicrobial and polymicrobial cultures were incubated for 16 h at 37°C with shaking at 200 rpm.

### Kinetic *agr* activation assay.

S. aureus(pDB22) was grown overnight in MOPS-buffered 0.6× TSB-g (pH 5.5) to inactivate *agr*. Additionally, S. aureus (JE2) and C. albicans were grown overnight in MOPS-buffered 0.6× TSB-g (pH 7). Cultures were centrifuged at 4,000 rpm for 3 min. Supernatant from the S. aureus JE2 and C. albicans cultures were collected and filter sterilized. Cells from S. aureus(pDB22) grown at pH 5.5 were washed with PBS and adjusted to 1 × 10^10^ CFU/ml. Cells were added to 1 ml of 5× MOPS-buffered TSB-g (pH 7) to a final concentration of 2 × 10^8^ CFU/ml. Four milliliters of either S. aureus (JE2) spent medium, C. albicans spent medium, or fresh medium was added to the cells along with 10 μg/ml erythromycin (for plasmid maintenance). Cultures were incubated at 37°C with shaking at 200 rpm. At 20-min intervals, 100-μl aliquots were removed, and fluorescence was measured as described above. Experiments were repeated in triplicate, and results are expressed as the mean AFU ± SEM.

### Alpha-toxin ELISA.

S. aureus alpha-toxin was quantified by an enzyme-linked immunosorbent assay (ELISA), as described previously (17). Briefly, 96-well plates were coated with 0.1 μg/ml MEDI4893* diluted in coating buffer and incubated overnight at 4°C. All wash steps were carried out with PBS containing 0.05% Tween 20. The plates were washed and blocked with SuperBlock (Pierce) for 1 h at room temperature. Fifty microliters of diluted filter-sterilized supernatant (taken at 16 h p.i.) was added to wells, with serial dilutions of native alpha-toxin included as the standard curve. The plates were incubated for 1 h at room temperature and then washed. Affinity-purified rabbit polyclonal anti-alpha-toxin antibody (2 μg/ml) was added to wells, and the plates were incubated for 1 h at room temperature. The plates were washed, and then a 1:10,000 dilution of Affinipure horseradish peroxidase (HRP)-coupled goat anti-rabbit IgG detection antibody (Jackson Immuno Research) was added. The plates were incubated for 1 h at room temperature and washed. 3,3′,5,5′-Tetramethylbenzidine (TMB) substrate was added to wells, and color was allowed to develop in the dark for 10 min. One hundred microliters of ELISA stop solution (0.2 M H_2_SO_4_) was added to each well, and wells were read at 450 nm using a spectrophotometer (Synergy, Biotek). Culture supernatants from S. aureus NE1354 Δ*hla* were used as background controls and were subtracted from sample wells to exclude any nonspecific binding of antibody by protein A. The experimental values were extrapolated to the standard curve. Experiments were completed in triplicate and shown as means ± SEM.

### CFU analysis.

CFU enumeration was done by serial plating of culture media onto YPD containing 20 μg/ml nafcillin (for *Candida* enumeration) and TSA containing 2.5 μg/ml amphotericin B (for S. aureus enumeration) via the drop-plate method (48). The plates were incubated overnight at 37°C, and the bacteria were enumerated and expressed as CFU/milliliter. CFU values are representative of three independent repeats and represented as median ± SEM.
